# Expanding the Phenotypic Spectrum of SPG7 Rare Damaging Variants: Insights From a Hungarian Cohort

**DOI:** 10.1111/cge.14719

**Published:** 2025-02-20

**Authors:** Idris Janos Jimoh, Peter Balicza, Tamas Szlepak, Dora Csaban, Aniko Gal, Adrienn Geresi, Zoltan Grosz, Agnes Palasti, Judit Boczan, Peter Klivenyi, Maria Judit Molnar

**Affiliations:** ^1^ Institute of Genomic Medicine and Rare Disorders Semmelweis University Budapest Hungary; ^2^ Doctoral School of Interdisciplinary Medicine University of Szeged Szeged Hungary; ^3^ HUN‐REN Multiomics Neurodegeneration Research Group Hungarian Research Network Budapest Hungary; ^4^ Department of Neurology University of Debrecen Debrecen Hungary; ^5^ Department of Neurology University of Szeged Szeged Hungary; ^6^ HUN‐REN‐SZTE Neuroscience Research Group, Hungarian Research Network, Danube Neuroscience Research Laboratory University of Szeged Szeged Hungary

**Keywords:** carrier risks, epidemiology, inheritance, mitochondrial disease, paraplegin, phenotypic spectra, SPG7

## Abstract

Mitochondria‐associated paraplegin dysfunction is primarily linked to spastic paraplegia; however, genetic alterations in SPG7 have been associated with a broader spectrum of clinical symptoms. To identify disease‐causing variants in the SPG7 gene, 437 patients with spastic ataxia, mitochondrial dysfunction‐associated symptoms, or motoneuron lesions detected by EMG have been tested. We aimed to assess the clinical spectrum and determine the frequency of damaging variants within patient groups, particularly those less studied. Using ACMG criteria, we identified 10 pathogenic or likely pathogenic variants, 5 variants of uncertain significance with predicted damaging effects, and a probable risk factor variant in 58 patients. We identified 25 biallelic and 33 monoallelic cases. The most common variant was p. Leu78Ter (*N* = 23), followed by p. Ala510Val (*N* = 21). The point prevalence of SPG7‐associated conditions in Hungary in 2024 is 0.46 per 100 000. In addition to well‐characterized cohorts, SPG7 alterations were frequently identified in cohorts with multisystemic mitochondrial disease and lower motoneuron lesions. Multiple mtDNA deletions and histological abnormalities were consistently observed across all groups. In monoallelic cases, no evidence of a digenic effect involving AFG3L2 was found. Both autosomal dominant and recessive inheritance patterns were documented, with monoallelic cases typically presenting with a milder phenotype.

## Graphical abstract

Rare damaging SPG7 variants were detected in 58 patients with spastic paraplegia, ataxia, mitochondrial dysfunction, or motoneuron lesions. The cumulative prevalence of SPG7‐related conditions in Hungary was estimated. Phenotypic spectra and mitochondrial dysfunction were assessed in monoallelic and biallelic cases, contributing to the understanding of the disease.

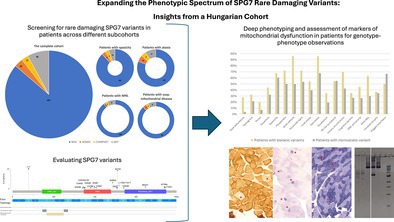

## 1 Introduction

The genetically heterogeneous hereditary spastic paraplegias (HSPs) can be classified into pure and complex forms [1]. In the pure form, spasticity is the most prominent feature, accompanied by spastic weakness of the lower limbs, and possibly impaired vibration sensation and urinary symptoms, without the presence of other neurological signs. In complex forms, additional neurological symptoms are present, such as neuropathy, seizures, cognitive impairment, and visual abnormalities, among others [2, 3]. The pathogenic variants of the SPG7 gene are associated with hereditary spastic paraplegia type 7 (HSP7). Initially, SPG7 was associated with pure HSP. After identifying a large cohort of patients with various SPG7 variants, the phenotypic spectrum was broadened [4] with ataxia, optic nerve impairment, and peripheral neuropathy. With the increasing accessibility of targeted NGS panel testing and whole‐exome sequencing (WES) it has been recognized that in a significant portion of SPG7 patients spasticity is not the first presenting symptom [5]. Even so, clinicians today frequently test for SPG7 only when spasticity is evident, leading to potential diagnostic challenges, delays, or misdiagnoses. The estimated prevalence of HSP7 is approximately 1.55%–12% among all HSP cases [1, 6]. The global prevalence of all SPG7‐associated conditions in the Caucasian population is estimated to be 0.22–0.72 per 100 000 [5, 7], but it might be higher due to variable expressivity and the broad phenotypic spectrum.

Additionally, it is important to note that while SPG7 variants are generally associated with an autosomal recessive (AR) inheritance pattern, the dominant effect of certain variants has also been proposed [6, 8]. A small subset of SPG7 cases may be associated with large deletions or duplications of the SPG7 gene, estimated to be present in approximately 2% of cases [9]. Also, in a limited number of cases with monoallelic rare deleterious variants (RDVs), the involvement of digenic factors, such as AFG3L2, has been suggested [10, 11]. Further investigations are needed to understand the nature of SPG7 rare damaging variants (RDVs) as well as the prevalence and impact of SPG7‐related diseases. Our phenotypic selection of patients was aligned with the gene's physiological and molecular functions to expand the phenotypic spectrum, enhancing clinical diagnostic workflows and improving patient management.

## 2 Patients and Methods

### 2.1 Patient Selection

We included the following patients in our cohort: (1) patients with spastic paraparesis, presumably caused by a genetic etiology, (2) patients with possibly inherited ataxia, (3) patients with lower motoneuron lesions detected by electromyography without marked amyotrophy, and (4) patients with suspected mitochondrial disorder involving the CNS (mt‐cohort). In this mt‐cohort, some patients presented external ophthalmoplegia (PEO), optic atrophy, muscle histology indicating mitochondrial dysfunction, or abnormal lactate stress tests suggestive of aerobic metabolic dysfunction. The presence of acquired neurological diseases was an exclusion criterion. For patients with suspected mitochondrial disease, multisystemic involvement was not used as an exclusion criterion. All participants provided written consent and were examined by a certified neurologist or clinical geneticist. Ashworth Spasticity grading, manual muscle strength scale, and SARA scoring were performed on patients carrying SPG7 variants as part of the deep phenotyping process.

Altogether, 437 probands (mean age: 53.22 years, ±15.24 years) were selected, including 197 females and 240 males. Spasticity was present in 267 patients (mean age: 53.16 years, ±15.11 years); 182 had ataxia as the presenting symptom (mean age: 54.67 years, ±14.44 years); 116 were referred due to suspected lower motoneuron disease or electromyography‐identified motoneuron lesions without amyotrophy (mean age: 55.74 years, ±15.13 years); and in 113 cases, mitochondrial disease was suspected (mean age: 54.95 years, ±14.58 years). Some patients exhibited more than one symptom or phenotypic feature at the time of examination, leading to their inclusion in multiple subcohorts. Consequently, 214 patients were counted in more than one cohort.

### 2.2 Methods

#### 2.2.1 Laboratory, Electrophysiological, and Imaging Investigations

Detailed laboratory examinations were performed on all patients. Cranial MRI and ENG/EMG investigations were conducted in all patients. DaTscan was performed on patients with a parkinsonian‐like phenotype.

#### 2.2.2 Genetic Testing

The entire coding region and exon/intron boundaries and neighboring nucleotides of the SPG7 gene were analyzed either by Sanger sequencing with an ABI Prism 3500 DNA Sequencer (Applied Biosystems, Foster City, USA) or with NGS targeted panel testing covering genes associated with ataxia, HSP, and ALS. Libraries of sheared genomic DNA were prepared for panels targeting either hereditary cerebellar ataxia or HSP genes. These were captured using custom‐designed probe sets to cover all coding and surrounding regions of SPG4, SPG7, and SPG11. Genomic DNA library preparation was performed using Agilent SureSelectQXT Human All Exon v5 reagents and SureSelectQXT Target Enrichment for Illumina Multiplexed Sequencing (Agilent Technologies, Santa Clara, CA, USA), following the manufacturer's protocol. Library preparation was followed by NGS using the Illumina HiSeq PE Cluster Kit v4 for cluster generation on the cBot, HiSeq SBS Kit v4 for sequencing on the HiSeq2500 system, and MiSeq Reagent Kit v2 (300‐cycles) for sequencing on the MiSeq (Illumina, San Diego, CA, USA).

Probands with monoallelic heterozygous variants underwent personalized extended NGS panel or WES to search for possible intergenomic interactions or other potentially disease‐causing damaging rare variants. In addition to the analysis of the targeted NGS panel, MLPA testing (Probemix P213‐B3) was conducted to detect large deletions in the SPG7 gene and targeted sequencing of AFG3L2 (RefSeq: NM_006796.3) exons (3, 7, 11, and 14–16) with potential digenic effect [10, 11, 12] was performed (*n* = 25) in monoallelic patients. Additional long‐range polymerase chain reaction (PCR) was performed on all patients to detect possible secondary mtDNA deletions.

#### 2.2.3 Bioinformatics Analysis

Variant calling from the NGS data was performed using GATK HaplotypeCaller (version 3.3‐0) following the GATK Best Practices Guidelines [13]. Variant Call Format (VCF) files were annotated with the SnpEff software [14] and the ClinVar database [15]. Variant filtering of exome sequencing data was carried out using the Franklin Genoox software and VariantAnalyzer software developed by the Budapest University of Technology and Economics. The nature of the novel alterations was determined according to the American College of Medical Genetics and Genomics (ACMG) guidelines [16, 17]. Where available, segregation analysis was performed. Key and selected factors considered during interpretation are outlined in Table 1. Additionally, other ACMG scoring factors, though not explicitly highlighted, were integrated during the interpretation.

**TABLE 1:** The identified RDVs in our cohort.

Variant	Exon	Mutation type	CADD	GERP	ACMG	GnomAD 2.1 all AF	All affected cases/families	Bi/monoalleic cases	Bi/monoallelic cases with spasticity	Bi/monoallelic cases with ataxia	Bi/monoallelic cases with LMNL	Bi/monoallelic cases with mitochondrial dysfunction	Asymptomatic carriers
p.Leu78Ter	2	Nonsense	33	5.32	PATH	0.00039	23/18	14/9	11/7	14/9	1/0	2/2	1
p.Lys340Glu	8	Missense	25.9	5.85	VUS	0	1/1	0/1	0/0	0/1	0/0	0/1	1
p.Gly344Asp	8	Missense	25.7	5.85	LP	0.00001	2/1	1/1	1/1	1/1	0/0	0/0	0
p.Gly352AlafsTer87	8	Frameshift	NA	5.85	LP	0	1/1	1/0	1/0	1/0	0/0	0/0	0
p.Gly352Ser	8	Missense	29.4	5.85	LP	0.00001	1/1	1/0	0/0	1/0	0/0	0/0	0
p.Val379Met	8	Missense	27.9	5.85	VUS	0.00003	1/1	0/1	0/1	0/1	0/0	0/0	0
p.Arg398Ter	9	Nonsense	41	5.63	PATH	0.00002	2/2	2/0	1/0	2/0	1/0	2/0	0
p.Tyr406Cys	9	Missense	29.5	5.63	LP	0.00001	1/1	0/1	0/1	0/1	0/0	0/0	0
p.Arg486Gln	11	Missense	22.6	5.42	RISK FACTOR	0.004722	5/5	0/5	0/2	0/4	0/1	0/0	0
p.Gln507Ter	11	Nonsense	49	5.42	PATH	0	1/1	1/0	0/0	1/0	0/0	0/0	0
p.Ala510Val	11	Missense	27.8	5.42	PATH	0.00289	21/19	12/9	9/5	12/7	1/2	2/2	3
c.1552 + 1G>T	11	Splice	34	5.42	PATH	0.00002	4/4	2/2	1/1	2/1	1/1	0/0	0
p.Val540Met	12	Missense	21.6	5.47	VUS	0.00002016	0/0	0/0	0/0	0/0	0/0	0/0	0
p.Ser645Thr	14	Missense	14.82	5.93	VUS	0.00076	2/2	0/2	0/1	0/2	0/0	0/1	0
p.Asn739fs	17	Frameshift	NA	5.59	PATH	0.00001	2/2	2/0	1/0	2/0	1/0	2/0	0
p.Tyr740Cys	17	Missense	25.7	5.59	VUS	0.00004599	1/1	0/1	0/0	0/1	0/0	0/0	0

*Note*: The table provides an overview of RDVs identified in the cohorts, highlighting key, but selected data relevant to their interpretation. It highlights information on the exon topology and mutation type, predictors of conservation and in silico pathogenicity scores—from which CADD and GERP scores are demonstrated—, allele frequencies from the gnomAD v2.1 population database. Additionally, the table presents information on total cases, affected families, and the distribution of biallelic and monoallelic cases.

Abbreviations: Variant type STOP‐nonsense—FRAME, frameshift; Miss, missense; SPLICE, splice site variants. ACMG classifications—AC, Allele count, AF, allele frequency; CompHet#, compound heterozygous patients to the variant; Het#, number of symptomatic carriers; Homo#, number of homozygous patients; LP, likely pathogenic; PATH, pathogenic; VUS, variant with unknown significance.

#### 2.2.4 Statistical Analyses

Statistical analysis was performed using Prism GraphPad V7.0b and SigmaPlot (2015) software. One‐way analysis of variance was used for multiple group comparisons, and independent samples *t* tests and *χ* ^2^ tests were used to compare two groups.

## 3 Results

**FIGURE 1:**
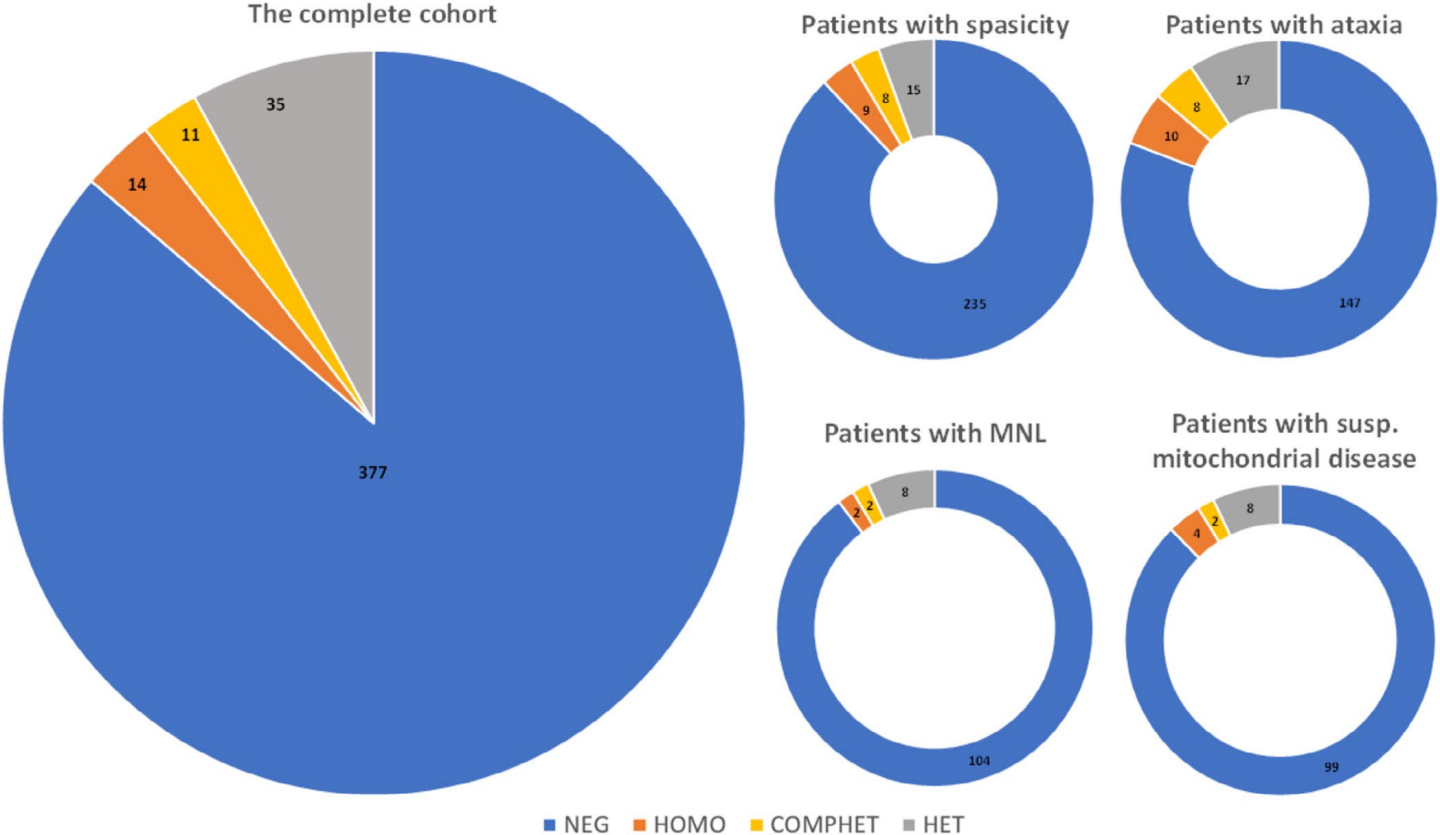
Prevalence of rare damaging variants in patients with spasticity, ataxia, motoneuron lesion (MNL), patients with suspected mitochondrial disorders. The thickness of the circle corresponds to the size of the cohorts. COMPHET, patients with compound heterozygous variants; HET, patients with heterozygous variant; HOMO, patients with homozygous SPG7 RDV; NEG, patients without SPG7 RDV.

At disease onset, the most common presenting symptoms were ataxia, paraparesis, dysarthria, or spasticity. In 13.5% (50/437) of the entire cohort (excluding probands' relatives), an SPG7 RDV was identified. The prevalence of RDVs in the four—partly overlapping—cohorts was as follows: the highest diagnostic success was observed in the ataxia cohort (Figure 1). Among patients with biallelic RDVs, 48% presented with paraparesis, 48% with ataxia, and 12% with visual complaints at the onset of their disease. In patients with monoallelic RDVs, 36% presented with spasticity, 21.4% with paraparesis, 35.7% with ataxia, 3.6% with visual complaints, and 14.2% with fasciculations or mitochondrial encephalomyopathy‐associated symptoms such as myalgia, fatigue, exercise intolerance, or PEO as the most prominent presenting symptoms. The prevalence of RDVs in each individual cohort is shown in Figure 1. The presence of damaging RDVs was most abundant in the ataxic and spastic cohorts with biallelic variants. The mitochondrial cohort had a higher prevalence of monoallelic RDVs compared to the other groups. In the remaining patients with monoallelic and biallelic variants, later examinations revealed early signs of corticospinal tract damage, which eventually led to spasticity or ataxia.

### 3.1 The SPG7 Variants

**FIGURE 2:**
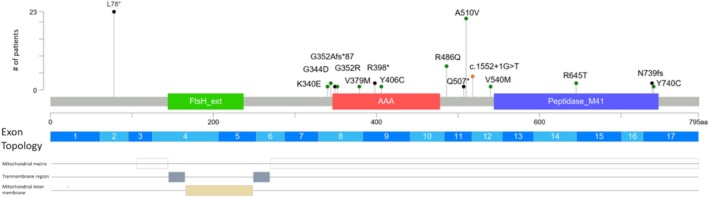
Lolliplot of detected SPG7 RDVs. The height of the lolliplot reflects the number of identified alleles, while the color indicates the variant type: Green: missense, black: frameshift/nonsense, orange: splice site. Variant locations are visualized in three layers: Essential functional domains of paraplegin encoded by SPG7: filamentation temperature‐sensitive mutant domain (FtsH), ATPase domain (AAA), and the Peptidase M41 domain. Exonic or genomic mapping: Dark and light blue bars indicate the corresponding exon/genomic location for each variant. Mitochondrial domain mapping: The bottom section highlights mitochondrial localization, showing that, apart from the L78 truncating variant, all other variants are located in the mitochondrial matrix.

In the SPG7 gene 16 rare RDVs were detected in 58 symptomatic cases. Of these, 36 cases carried a missense variant (41 alleles), 26 cases nonsense variants (35 alleles), 3 cases frameshift variants (3 alleles), and 4 cases carried splice‐site variants (4 alleles). MLPA did not reveal any deletions/duplications in the SPG7 gene. According to the ACMG guidelines, 10 rare variants were considered pathogenic or likely pathogenic. In addition to the clearly clinically significant variants, several VUS were identified. Among these, five variants were considered to be a RDV based on in silico predictions and additional ACMG criteria, which suggested a potential deleterious effect on protein function. Consequently, patients harboring these RDVs were included in our cohort even though, these variants did not meet the criteria for a “likely pathogenic” or “pathogenic” classification according to the ACMG guidelines. Additionally, we identified the p. Arg486Gln variant in five cases in heterozygous form. This rare variant is considered a genetic risk factor for ALS [18]. In our cohort, the p. Leu78Ter (*N* = 23) and p. Ala510Val (*N* = 21) RDVs were the most prevalent disease‐causing variants (Figure 2) [19]. The p. Leu78Ter variant, the most common in the entire cohort, was present in 23 out of 58 symptomatic patients, either in a heterozygous (*N* = 9), compound heterozygous (*N* = 5), or homozygous (*N* = 9) form. The second most common variant was p. Ala510Val, found in 21 out of 58 affected cases, present in homozygous form (*N* = 5), compound heterozygous form (*N* = 7), and heterozygous form (*N* = 9). The other RDVs had significantly lower incidence, and certain variants have also been identified in asymptomatic carriers. Table 1 demonstrates information on all cases, affected families, and the distribution of biallelic and monoallelic cases in patient subgroups, highlighting that certain variants were identified in both monoallelic and biallelic forms, while others were exclusive to only one type of zygosity.

### 3.2 Clinical Phenotype of Patients With Identified SPG7 RDV

We identified 23 families (23 probands) with biallelic SPG7 RDVs, 22 symptomatic patients with monoallelic variants, and 5 carrying a genetic risk factor for ALS. Family cascade testing identified two additional symptomatic relatives with homozygous RDVs, six symptomatic monoallelic SPG7 RDV carriers, and five asymptomatic relatives with monoallelic RDVs. Most of the asymptomatic relatives were relatively young (30.75 ± 2.9 years). Overall, we identified 58 cases carrying at least one RDV or a risk factor allele and exhibiting neurological symptoms (Supporting Information Table). Among the identified patients, 14 were homozygous, 11 were compound heterozygous, and 28 were heterozygous for RDVs. Additionally, five patients carried a risk factor allele. The average age at onset in affected individuals was as follows: 41.12 (±9.97) years for patients with biallelic variants (39 ± 9.73 years for homozygous, 44.3 ± 9.95 years for compound heterozygous), 45.86 (±13.37) years for monoallelic heterozygous patients, and 51.6 (±19.65) years for patients with the risk allele.

### 3.3 Brain Imaging

The MRI and imaging findings in the patients were consistent with previous studies in terms of cerebellar volume and T2 signals. The prevalence of cerebellar atrophy was 36.84% (19/52) in the biallelic cases. In addition to cerebellar atrophy, the following findings were identified in our patients' brain MRIs: dentate nuclei hyperintensity, nonspecific focal, and confluent T2 white matter hyperintensity. Additionally, we identified the “ears of the lynx” sign in one of our patients, which has not previously been associated with SPG7‐related disorders but is frequently observed in SPG11 patients [20]. These findings were not specific to a particular variant, variant type, or zygosity. Some patients with atypical parkinsonism underwent DaTscan, and none of them showed impaired dopaminergic function.

### 3.4 Genotype–Phenotype Correlations

**FIGURE 3:**
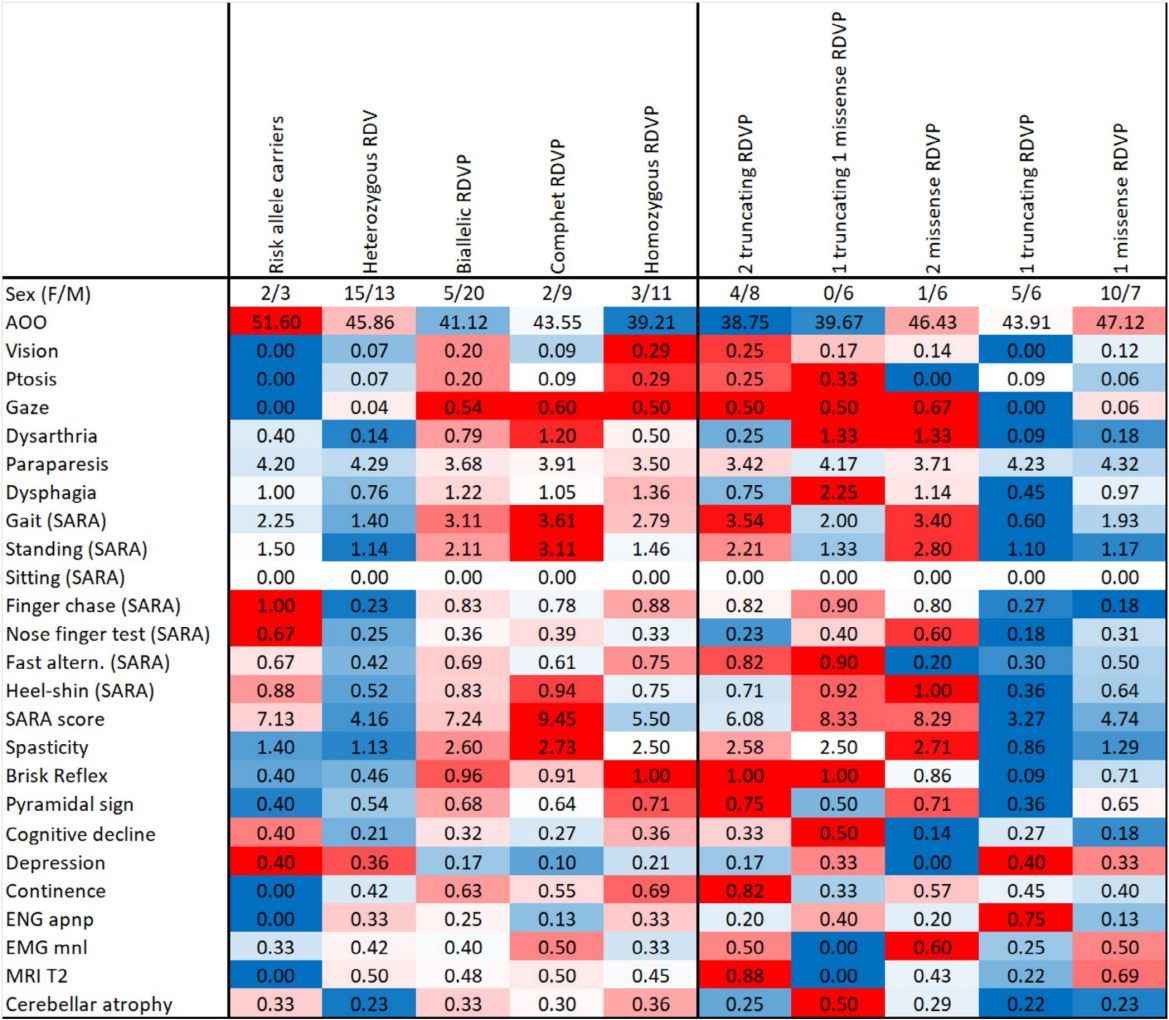
Genotype–phenotype observations. The figure displays the prevalence of key SPG7‐associated features across genotype groups, with columns representing genotypes and rows denoting associated clinical features. The genotype groups include zygosity‐based groups (heterozygous, biallelic, and compound heterozygous patients) on the left side of the figure. A mutation type‐based categorization (patients with RDV [RDVP] are the following: two truncating RDVP, one truncating and one missense RDVP, two missense RDVP, one truncating RDVP, and one missense RDVP) is provided on the right side of the figure. The means of SARA subscores are indicated with “(SARA).” Heatmap colors are scaled row‐wise to highlight the genotype group most associated with each feature. AOO, age of onset; DD, disease duration; EMG mnl, motor neuron lesions detected by electromyography; ENG apnp, axonal polyneuropathy identified via electroneurography; Fast altern., fast alternating movements; Mito involvement, mitochondrial dysfunction evidenced by muscle histology or mtDNA multiplex deletions. MRI T2, T2 hyperintense foci observed on MRI.

The type and location of the SPG7 variant did not significantly affect the age of onset or the symptoms. However, homozygous and compound heterozygous patients appeared to differ in terms of clinical phenotype, particularly in the severity of spasticity, muscle strength, and SARA scores. Monoallelic patients exhibited similar symptoms to those with biallelic variants, but they tended to have a milder phenotype (Figure 3). Although multiple investigations, besides the previous observation we did not find strong evidence regarding the correlation between the mutation type, age of onset, or symptom severity.

Atypical Parkinsonian features were observed in three biallelic and two monoallelic patients. P4 presented with paraparesis and ataxia, later developing freezing and gait difficulties. P11C initially had classical HSP but later exhibited hypomimia, bradykinesia, and Parkinsonian gait. P19 showed rigidity, static tremor, bradykinesia, cognitive decline, and spastic ataxia. P29 besides cerebellar ataxia as the leading symptom, displayed upper limb tremor, rigidity, and Parkinsonian gait. P42 had symmetric tremor, rigidity, hypokinesis, and pyramidal signs alongside mild lower limb paresis and gait instability. Patients did not respond to dopaminergic drugs.

### 3.5 Evidence of Mitochondrial Involvement

**FIGURE 4:**
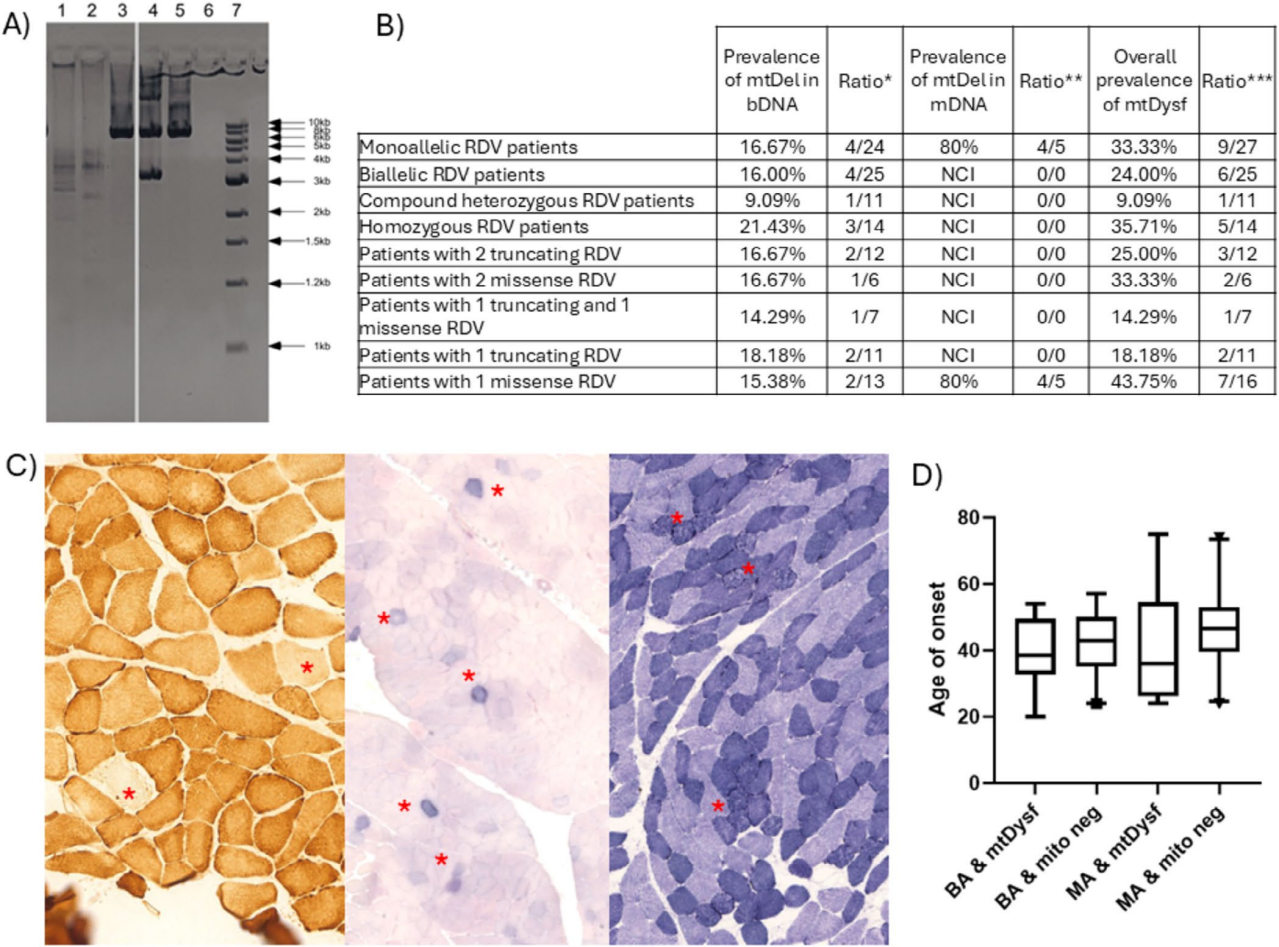
Investigations for mitochondrial involvement. (A) Representative image of long‐range PCR results: 1 and 2. Patient with multiple mtDNA deletions; 3. Patient without mtDNA deletions; 4. Control for single mtDNA deletion; 5. Control for wild‐type mtDNA; 6. No template control; 7. Molecular‐weight size marker. (B) Deletion results of patients. BDNA, sDNA: DNA samples isolated from blood or skeletal muscle. Ratio*: Positive cases for multiple mtDNA deletions in bDNA divided by all bDNA cases. Ratio**: Positive cases for multiple mtDNA deletions in mDNA/all mDNA cases. NCI: No investigation was conducted for cases that met the criteria. mtDysf: Patients with mitochondrial dysfunction identified by either muscle histology or mitochondrial deletion analysis. Ratio***: Cases with mtDysf divided by all cases investigated for mtDysf. (C) Muscle histologic images from the left: COX‐negative fibers (P38); Ragged blue fibers (P46); subsarcolemmal granular aggregations on NADH staining (P11C). (D) Age of onset distribution among patient subgroups. BA, biallelic RDV patients; MA, monoallelic RDV patients; mito neg, patients with no histology or deletion analysis proven mitochondrial dysfunction.

In the mitochondrial cohort, most patients (77 cases) had muscle biopsy. Among these, seven patients carried SPG7 RDVs. The following patients had mitochondrial dysfunction associated with ragged blue fibers in their muscle biopsies: P4 and P11C with homozygous p.Leu78Ter, P32 and P46 with heterozygous p.Ala510Val, P38 with heterozygous p.Ser645Thr, P48 with heterozygous p.Val540Met, and P49 with heterozygous c.1552 + 1G>T. Muscle histology provided supportive evidence of the damaging effect of the p.Ser645Thr variant, but did not conclusively establish its pathogenicity. While the variant remains classified as VUS, the investigation identified a feature commonly observed in SPG7 patients [3, 4]. Additional mtDNA deletion analysis was conducted on patients, using 52 blood‐derived and 6 muscle‐derived DNA samples (Figure 4). Multiple mtDNA deletions were found in 15.38% of blood samples (8/52) and 66% of muscle samples (4/6). Combining histological findings with mtDNA data, mitochondrial dysfunction was detected in 33.3% of heterozygous and 16.0% of biallelic patients. Statistical analyses showed no significant impact of mtDNA deletions (*t* test, *p* = 0.1213) or their combination with zygosity (one‐way ANOVA, *p* = 0.1575) on the age of onset.

## 4 Discussion

This is the first paper focusing on Hungarian patients with SPG7 RDVs. The cumulative point prevalence of SPG7‐associated conditions in Hungary is 5.1 per 1 million (0.05 per 10 000). Although SPG7‐associated conditions appear to be very rare, SPG7 is one of the most commonly altered nuclear mitochondrial [21] genes. Paraplegin is expressed in a wide variety of cells in the central nervous system [22, 23] which may contribute to the colorful phenotypic spectra.

Corticospinal tract impairment, leading to spastic paraparesis, is a common consequence of SPG7 RDVs and the hallmark symptom of hereditary spastic paraplegia type 7 (HSP7) [24] While patient phenotypes can evolve over time, spasticity was absent in 8% of individuals with biallelic RDVs and 40% with monoallelic RDVs during follow‐up. The pathomechanism underlying corticospinal tract damage resembles upper motor neuron lesions, similar to those in ALS.

In our cohort, 3.4% of patients with suspected motor neuron disease (without amyotrophy) had biallelic SPG7 RDVs, and 4.3% had monoallelic RDVs. EMG identified motor neuron lesions in 40% of monoallelic and 55% of biallelic RDV cases.

Although the p.Arg486Gln variant is classified as benign under ACMG guidelines, it has been suggested as an autosomal dominant risk [3, 18] or modifying factor for ALS [25]. Among five patients carrying this variant in heterozygous form, two were referred to us with suspected ALS. These patients exhibited diverse phenotypes, often with late onset, including ALS‐like features and SPG7‐related symptoms such as spastic ataxia. However, the role of this variant as a risk factor remains uncertain and requires further study.

Less severe SPG7 variants, alongside pathogenic ones, may contribute to ALS or motor neuron syndromes. Indeed, SPG7 mutations have been implicated in 7% of slowly progressive upper motor neuron syndromes [26]. Diagnosing HSP versus ALS is particularly challenging when patients with paraparesis undergo EMG without visible amyotrophy, a feature typically absent in early ALS [27]. Findings such as denervation or neurogenic lesions can mimic early ALS, emphasizing the need for SPG7 genetic screening in these cases.

Paraplegin's role in energy production in Purkinje cells and cerebellar nuclei links SPG7 RDVs to their degeneration. This dysfunction manifests in imaging and clinical signs, including cerebellar ataxia. In our cohort, cerebellar atrophy was observed in 33% of biallelic and 26.3% of monoallelic RDV patients. A recent study also reported increased T2‐weighted MR hyperintensity in cerebellar dentate nuclei among SPG7 patients [28]. In our cohort, 45.5% of patients with biallelic RDVs and 57.9% of patients with monoallelic RDVs had T2 hyperintense white matter foci on T2‐weighted MR images, making it a frequent but nonspecific feature. Brain atrophy and cognitive impairment have also been reported in patients with SPG7 RDVs [29, 30] several years after the onset of the disease, which aligns with our observations.

Interestingly, five of our patients exhibited Parkinsonian‐like phenotypic features. In a limited cohort, it has been proposed that paraplegin is expressed in the globus pallidus within the basal ganglia [31, 32]. Patients presenting Parkinsonian‐like phenotypes did not show a response to dopamine agonists. All patients tested negative for the most common monogenic Parkinsonian syndromes. Additionally, in patients who underwent DaTscan (*n* = 2), no dopaminergic dysfunction was detected. Thus, Parkinsonian‐like features may represent a rare and interesting manifestation of SPG7 RDVs.

Although SPG7 is generally considered an AR disorder, we propose that certain SPG7 RDVs in monoallelic form may be associated with symptoms. Information on patients with heterozygous SPG7 variants is limited. According to literature, SPG7‐associated disease may originate from digenic variants. Among the potential interacting genes, AFG3L2 emerges as the most convincing candidate, which has been implicated in several phenotypes. Notably, digenic cases involving SPG7 and AFG3L2 variants have been reported but, none of our patients harbored secondary AFG3L2 variants. Consequently, the presence of digenic factors exists, but based on literature and our observations, it is unable to fully explain the widespread occurrence of monoallelic presentations.

The abundance of heterozygous patients in our subcohorts, may be related to the molecular function of paraplegin. Paraplegin is involved in mitochondrial quality control, ribosome assembly, and OXPHOS biogenesis [33]. The decreased activity of paraplegin impairs respiratory complex I and increases sensitivity to reactive oxygen species [3, 4]. Some other nuclear‐encoded mitochondrial genes (e.g., POLG1, TWNK) have variant‐specific inheritance patterns and clinical spectra. Thus the physiological function may explain the proposed AD effects of SPG7 [5, 6, 8, 34]. Based on segregation and phenotypic data, the presence of mitochondrial dysfunction, and MRI features in patients having monoallelic SPG7 RDVs, the p.Ala510Val, and p.Leu78Ter variants showed the most reliable dominant or semi‐dominant effects, in our cohort. While our findings do not establish definitive evidence of dominant inheritance, they further support the notion that monoallelic variants in SPG7 may independently contribute to pathogenicity. Based on our observations, truncating variants may be particularly likely to cause disease in a monoallelic form due to their more severe impact on protein function. We observed 11 patients with monoallelic truncating variants, with the p. Leu78Ter variant being the most common. However, the missense p. Ala510Val variant was also frequently observed in monoallelic form, potentially due to its specific location. Additionally, environmental factors such as medications, infections, or alcohol‐related mitochondrial dysfunction might influence the onset of the disease and the penetrance of certain variants. This influence of environmental factors has been noted in mitochondrial disorders. Patients with monoallelic RDVs generally exhibited a relatively milder phenotype, similar to the pattern seen in POLG‐associated disorders. Further investigations are needed to elucidate the penetrance and expressivity of such variants. Our findings align with previous studies on SPG7‐related disorders, further emphasizing the link between SPG7 RDVs and mitochondrial dysfunction. Mitochondrial dysfunction has been identified in patients who were not initially suspected of having mitochondrial disease. As therapeutic trials for mitochondrial disorders advance, demonstrating evidence of mitochondrial dysfunction is likely to become a critical component of clinical practice in the future.

### 4.1 Limitations of the Study

The prevalence of the two variants (p. Leu78Ter and p. Ala510Val) and the number of patients affected by SPG7 RDVs did not provide sufficient data for precise variant‐based predictions. Calculating the penetrance of monoallelic variants proved challenging due to several factors: the limited willingness of probands and their relatives to undergo segregation analysis, the relatively young age of many relatives, and the reluctance of carriers to participate in further investigations. Additionally, the retrospective design of the study complicated recontacting patients, limiting the availability of certain sample types.

While our cohort is sizeable and represents a clinically relevant population, several factors constrain the evaluation of polygenic risks and other potential contributors. These include the recruitment focus on specific patient groups, the lack of inclusion of healthy elderly individuals, and the methodologies employed.

## 5 Conclusion

Rare damaging variants in SPG7 are disease‐causing genetic alterations, even in cases where classical spasticity is not a prominent feature. SPG7 variants are associated with a spectrum of mitochondrial disorder phenotypes, including progressive external ophthalmoplegia, and myopathy. Genetic testing for SPG7 should be considered in the differential diagnosis of lower motor neuron lesions, especially when amyotrophy is absent.

The p.Leu78Ter variant is the most common disease‐causing SPG7 variant in Hungary. Our findings suggest it may follow both autosomal dominant and autosomal recessive inheritance patterns. Patients with monoallelic p.Leu78Ter variants typically exhibit a milder phenotype and later onset compared with those with biallelic variants. The p.Ala510Val variant is the second most common SPG7 RDV and also shows evidence of autosomal dominant effects.

Although no RDVs were identified in AFG3L2 in monoallelic cases, the possibility of digenic effects involving SPG7 remains a compelling hypothesis. Further research is needed to clarify these interactions and their contribution to disease phenotypes.

## Ethics Statement

Patients provided written informed consent for publishing medical data per the Declaration of Helsinki, and the study was approved by the Hungarian Ethical Committee of the Medical Research Council (TUKEB 44599–2/2013/EKU 535/2013).

## Conflicts of Interest

The authors declare no conflicts of interest.

## Peer Review

The peer review history for this article is available at https://www.webofscience.com/api/gateway/wos/peer‐review/10.1111/cge.14719.

## Supporting information


**Data S1.** Supporting Information.

## Data Availability

The supporting Information table includes the shareable clinical data.
