# Supplementary material for: Expanding the Phenotypic Spectrum of SPG7 Rare Damaging Variants: Insights From a Hungarian Cohort
Source: Clin Genet. 2025 Feb 20;108(2):124–33. doi: 10.1111/cge.14719 (PMC12215234; doi:10.1111/cge.14719)
Supplement: Supplementary file 1 — Data S1. Supporting Information. [file CGE-108-124-s001.docx]

# Supplementary table

*3. TABLE - Phenotypic spectra of patients with pathogenic or likely pathogenic SPG7 variants – Asymptomatic carriers and patients with only risk alleles have been excluded from the table. Legend: PatientID is composed of an individual number assigned to each patient, with a familyID (a single letter) added to indicate kinship among patients within the cohort, when it was applicable., atx – ataxia, dysa – dysarthria, dem – dementia, PD – parkinsonian-like symptoms, fasc – fasciculations, spas – spasticity, mit – findings and symptoms related to mitochondrial encephalomyopathy, AOO- Age of onset, upar – upper limb paresis, para – paraparesis, DD – Disease duration, Vision – visual field impairment or scotomy, gaze- gaze palsy and/or ophtalmoplegia, Dysarthrtia according to scara score, paraparesis according to Oxford muscle strength scale, spasticity according to Asworth scale, incontinence: urge – urge urinarinary incontinence, rete – retention, cat – catheter, norm – no abnormality, electrophysiological findings: axpnp – axonal polyneuropathy, dpnp – demyelinating polyneuropathy, myo – myogenic lesion on electrophysiologic examination, mnl – motoneuron lesion on electrophysiologic examination, Imaging findings: Ca – cerebellar atrophy, Ba – cerebral atrophy, T2 – T2 enhancing lesions, GPI – basal ganglia hypodensity, nSPECT – normal SPECT, nDaT – normal DaTscan, Mito.Dysf – Investigation results for mitochondrial dysfunction; B – multiple mtDNA deletions detected in a blood-derived DNA sample; M – multiple mtDNA deletions detected in a muscle tissue-derived DNA sample; H – histological signs of mitochondrial dysfunction.*

| **PatientID** | **Homozygous variants** | **Sex** | **AOO / DD** | **Presenting symptom** | **Vision** | **Ptosis** | **Gaze** | **Dysarthria** | **Paraparesis** | **SARA score** | **Spasticity** | **Brisk Reflex** | **Pyramidal sign** | **Cognitive** | **Depression** | **Continence** | **Mito. Dysf.** | **ENG/EMG** | **Imaging** |
| --- | --- | --- | --- | --- | --- | --- | --- | --- | --- | --- | --- | --- | --- | --- | --- | --- | --- | --- | --- |
| 1a | p.Leu78Ter | F | 28/32 | Vision | ✓ | ✓ | ✓ | 0 | 2,5 | 12 | 3 | 3 | ✓ | Ø | Ø | rete, uri | Ø | mnl | no, T2, GPI, nSPECT |
| 2a | p.Leu78Ter | M | 44/7 | Atx | Ø | ✓ | ✓ | 2,5 | 5 | 9 | 2,5 | 2 | Ø | Ø | Ø | norm | Ø | mnl | T2 |
| 3 | p.Leu78Ter | M | 43/8 | Para | Ø | Ø | ✓ | 3,5 | 5 | 8,5 | 2 | 2 | ✓ | ✓ | ✓ | rete, uri | Ø | norm | ca, T2, Ba |
| 4 | p.Leu78Ter | M | 48/11 | Para, atx | Ø | Ø | Ø | 0 | 4 | 4 | 1 | 2 | ✓ | Ø | Ø | urge | H | norm | T2 |
| 5 | p.Leu78Ter | F | 20/42 | Vis, para | ✓ | Ø | Ø | 0 | 3 | 10 | 4 | 4 | ✓ | ✓ | ✓ | uri | B | aPNP, mnl | NA |
| 6 | p.Leu78Ter | M | 32/16 | Para, atx | Ø | Ø | Ø | 0 | 2 | 10,5 | 4 | 3 | ✓ | ✓ | Ø | rete, uri | Ø | mnl | NA |
| 7 | p.Leu78Ter | M | 46/21 | Para, vis | ✓ | Ø | ✓ | 3 | 3 | 14,5 | 4 | 3 | ✓ | Ø | Ø | urge | Ø | axpnp | vasc |
| 8 | p.Leu78Ter | M | 35/9 | Atx, pnp | Ø | Ø | Ø | 0 | 5 | 2 | 2 | 2 | ✓ | Ø | Ø | NA | Ø | NA | NA |
| 9b | p.Leu78Ter | F | 39/17 | Para, uinc | Ø | Ø | Ø | 0 | 1 | 1 | 3 | 2 | Ø | Ø | Ø | urge | B | axpnp | T2 |
| 10 | p.Ala510Val | M | 54/5 | Para, dysar | Ø | Ø | ✓ | 0 | 4 | 0 | 0 | 2 | ✓ | Ø | Ø | norm | Ø | norm | normal |
| 11c | p.Ala510Val | M | 38/19 | Para | Ø | ✓ | Ø | 3,5 | 3 | 17 | 4 | 3 | ✓ | Ø | ✓ | rete, uri | Ø | axpnp | ca, nDaT |
| 12c | p.Ala510Val | M | 54/5 | Spas, para | ✓ | ✓ | Ø | 2,5 | 4 | 9 | 4 | 2,5 | Ø | ✓ | Ø | rete, uri | B | axpnp | Ba |
| 13 | p.Ala510Val | M | 38/19 | Atx, dysar | Ø | Ø | ✓ | 2 | 5 | 8 | 2 | 2 | Ø | Ø | Ø | norm | Ø | NA | Ca, GPI |
| 14 | p.Ala510Val | M | 41/9 | Atx | Ø | Ø | ✓ | 2 | 5 | 5 | 2 | 3 | ✓ | ✓ | Ø | norm | Ø | norm | Ca, Ba |

| **PatientID** | **Compound heterozygous variants** | **Sex** | **AOO / DD** | **Presenting symptom** | **Vision** | **Ptosis** | **Gaze** | **Dysarthria** | **Paraparesis** | **SARA score** | **Spasticity** | **Brisk Reflex** | **Pyramidal sign** | **Cognitive** | **Depression** | **Continence** | **Mito.Dysf.** | **Electro-phys** | **Imaging** |
| --- | --- | --- | --- | --- | --- | --- | --- | --- | --- | --- | --- | --- | --- | --- | --- | --- | --- | --- | --- |
| 15 | p.Leu78Ter / p.Ala510Val | M | 36/11 | Spas, dysa | Ø | Ø | Ø | 2 | 4 | 13 | 4 | ✓ | ✓ | Ø | Ø | urge | Ø | mnl | norm |
| 16 | p.Gly352fs / p.Ala510Val | M | 57/6 | Atx, parap | Ø | Ø | ✓ | 0 | 3 | 9 | 4 | ✓ | Ø | Ø | Ø | urge | Ø | axpnp | T2, nSPECT |
| 17 | p.Gly352Ser / p.Gln507* | M | 50/8 | Atx, dysa | Ø | Ø | ✓ | 3 | 4 | 13 | 0 | ✓ | Ø | Ø | Ø | norm | Ø | mnl | ca, T2, nSPECT |
| 18 | p.Arg398Ter / p.Asn739fs | F | 42/12 | Atx, spas | Ø | Ø | ✓ | 0 | 4 | 12 | 1 | ✓ | Ø | Ø | Ø | urge | Ø | norm | NA |
| 19 | p.Asn739fs / p.Arg398Ter | M | 52/22 | Atx, spas | Ø | ✓ | ✓ | 0 | 4 | 18 | 4 | ✓ | ✓ | ✓ | Ø | rete, uri | Ø | axpnp, mnl | Ca, T2 |
| 20 | c.1552+1G>T / p.Ala510Val | M | 37/28 | Atx, dysa | ✓ | Ø | ✓ | 3 | 5 | 15 | 3 | ✓ | ✓ | Ø | Ø | urge | B | axpnp, mnl | Ca |
| 21 | c.1552+1G>T / p.Leu78Ter | M | 36/7 | Spas | Ø | Ø | Ø | 0 | 5 | 5 | 3 | ✓ | ✓ | Ø | Ø | norm | Ø | NA | T2 |
| 22d | p.Ala510Val / p.Gly344AsP | M | 24/13 | Spas | Ø | Ø | Ø | 4 | 4 | 11 | 3 | ✓ | Ø | ✓ | ✓ | norm | Ø | myo | cyst |
| 23 | p.Leu78Ter / p.Ala510Val | M | 45/8 | Atx, parap | Ø | Ø | NA | NA | 3 | 0 | 2 | ✓ | ✓ | ✓ | NA | norm | Ø | norm | norm |
| 24 | p.Leu78Ter / p.Ala510Val | F | 54/3 | Ataxia | Ø | Ø | Ø | 0 | 3 | 5 | 3 | Ø | ✓ | Ø | Ø | norm | Ø | NA | T2 |
| 25 | p.Leu78Ter / p.Ala510Val | M | 46/19 | Spas, para | Ø | Ø | ✓ | 0 | 4 | 3 | 3 | ✓ | ✓ | Ø | Ø | urge | Ø | NA | norm |

| PatientID | Monoalleic variants | Sex | AOO/DD | Presenting symptom | Vision | Ptosis | Gaze | Dysarthria | Paraparesis | SARA score | Spasticity | Brisk Reflex | Pyramidal sign | Cognitive | Depression | Continence | Mito.Dysf. | Electro-phys | Imaging |
| --- | --- | --- | --- | --- | --- | --- | --- | --- | --- | --- | --- | --- | --- | --- | --- | --- | --- | --- | --- |
| 26E | p.Leu78Ter | F | 48/10 | Spas, uincon | Ø | Ø | Ø | 0 | 3.5 | 2 | 3 | Ø | ✓ | Ø | ✓ | cat | Ø | NA | NA |
| 27E | p.Leu78Ter | F | 48/12 | Spas, atx | Ø | Ø | Ø | 0 | 5 | 0 | 1.5 | Ø | Ø | ✓ | ✓ | urge | Ø | NA | norm |
| 28. | p.Leu78Ter | F | 56/5 | Atx | Ø | Ø | Ø | 0 | 3 | 12 | 0 | Ø | ✓ | Ø | Ø | norm | Ø | apnp | Ca |
| 29. | p.Leu78Ter | M | 60/5 | Atx, mito | Ø | Ø | Ø | 0 | 4 | 12 | 2 | Ø | ✓ | ✓ | ✓ | rete | Ø | apnp | Ca, T2, nDaT |
| 30. | p.Leu78Ter | M | 45/23 | Atx, Dem, PD | Ø | Ø | Ø | 0 | 5 | 2 | 0 | Ø | Ø | ✓ | Ø | urge | Ø | NA | vasc |
| 31C | p.Ala510Val | M | 24/10 | Spas | ✓ | Ø | Ø | 0 | 4 | 3.5 | 1.5 | Ø | Ø | Ø | Ø | norm | Ø | NA | NA |
| 32. | p.Ala510Val | F | 50/8 | Fasc, mito | Ø | Ø | Ø | 0 | 5 | 6 | 0 | ✓ | ✓ | Ø | ✓ | norm | H | norm | norm |
| 33. | p.Ala510Val | F | 64/6 | Atx | Ø | Ø | Ø | 0 | 5 | 4 | 2 | ✓ | ✓ | Ø | ✓ | norm | B | NA | NA |
| 34. | p.Ala510Val | F | 61/7 | Dem | Ø | Ø | Ø | 0 | 5 | 3 | 0 | Ø | Ø | ✓ | ✓ | norm | Ø | NA | Ca, Ba |
| 35. | c.1552+1G>T | M | 53/8 | Atx, spas | Ø | Ø | Ø | 0 | 3 | 5 | 2 | ✓ | ✓ | Ø | NA | urge | Ø | apnp | T2 |
| 37. | p.Lys340Glu | M | 50/4 | Vision, atx | Ø | Ø | Ø | 0 | 4 | 7 | 3 | ✓ | ✓ | ✓ | ✓ | cat | B | a+dPNP | T2, Ca |
| 38. | p.Ser645Thr | F | 38/1 | Mito, atx | Ø | Ø | Ø | 0 | 2.5 | 8 | 1 | ✓ | Ø | ✓ | Ø | urge | H,M,B | mnl | T2 |
| 39. | p.Ser645Thr | M | 41/2 | Spas | Ø | Ø | Ø | 0 | 2 | 14 | 2.5 | ✓ | ✓ | Ø | Ø | rete | Ø | apnp | T2 |
| 40D | p.Gly344AsP | F | 25/28 | Spas | Ø | Ø | Ø | 0 | 5 | 0 | 2 | Ø | Ø | Ø | NA | NA | Ø | NA | NA |
| 41. | p.Val379Met | M | 45/1 | Atx | ✓ | Ø | Ø | 0 | 5 | 10 | 0 | ✓ | ✓ | Ø | Ø | norm | Ø | NA | T2 |
| 42. | p.Tyr406Cys | M | 75/2 | PD | Ø | Ø | Ø | 0 | 4 | 4 | 1 | ✓ | ✓ | Ø | Ø | urge | Ø | NA | norm |
| 43. | p.Ala510Val | F | 42/3 | Spas | Ø | Ø | Ø | 0 | 5 | 2 | 3 | ✓ | ✓ | Ø | NA | NA | B | NA | T2 |
| 44. | p.Ala510Val | F | 34/2 | Fasc, parap | Ø | Ø | Ø | 0 | 4 | 2 | 0 | ✓ | ✓ | Ø | Ø | norm | NA | mnl, apnp | T2 |
| 46. | p.Ala510Val | M | 38/2 | Dysa, mito, atx | Ø | ✓ | Ø | 1 | 4 | 9 | 3 | ✓ | ✓ | Ø | ✓ | urge | M,H | NA | T2 |
| 47. | p.Tyr740Cys | F | 30/28 | Dysa | Ø | Ø | Ø | 1 | 5 | 3 | 0 | ✓ | Ø | Ø | Ø | norm | Ø | norm | Ba, Ca, T2 |
| 48. | p.Val540Met | F | 24/4 | Dysp, parap, fasc, mit | Ø | Ø | ✓ | 1 | 4 | 4 | 1 | Ø | ✓ | Ø | Ø | norm | M,H | mnl | T2 |
| 49. | c.1552+1G>T | M | 36/1 | Fasc, dem, Uparesis | Ø | ✓ | Ø | 1 | 3 | 3 | 1 | Ø | Ø | Ø | ✓ | norm | Ø | aPNP, mnl | norm |
| 50B | p.Leu78Ter | F | 39/11 | Parap | Ø | Ø | Ø | 0 | 5 | 0 | 0 | Ø | Ø | Ø | Ø | norm | B | NA | NA |
| 51B | p.Leu78Ter | F | 38/2 | Parap | Ø | Ø | Ø | 0 | 5 | 0 | 0 | Ø | Ø | Ø | Ø | norm | Ø | NA | norm |
| 52. | p.Leu78Ter | M | 58/9 | Parap | Ø | Ø | Ø | 0 | 5 | 0 | 0 | Ø | Ø | Ø | Ø | norm | B | NA | norm |
| 53. | p.Ala510Val | M | 28/3 | Mit | Ø | Ø | Ø | 0 | 5 | 0 | 0 | Ø | Ø | Ø | Ø | norm | Ø | NA | NA |
| 54A | p.Leu78Ter | M | 37/1 | Spas | Ø | Ø | Ø | 0 | 5 | 0 | 0 | Ø | Ø | Ø | Ø | norm | Ø | NA | norm |
| 55. | p.Ala510Val | F | 50/12 | Parap, spas | Ø | Ø | Ø | 0 | 5 | 1 | 2 | ✓ | ✓ | Ø | Ø | urge | Ø | mnl | norm |

| PatientID | RISK FACTOR VARIANT | Sex | AOO/DD | Presenting symptom | Vision | Ptosis | Gaze | Dysarthria | Paraparesis | SARA score | Spasticity | Brisk Reflex | Pyramidal sign | Cognitive | Depression | Continence | Mito.dysf. | Electro-phys | Imaging |
| --- | --- | --- | --- | --- | --- | --- | --- | --- | --- | --- | --- | --- | --- | --- | --- | --- | --- | --- | --- |
| 58. | p.Arg486Gln | M | 42/9 | Atx | Ø | Ø | Ø | 0 | 4 | 6 | 2 | ✓ | ✓ | ✓ | Ø | norm | NA | NA | Ca |
| 60. | p.Arg486Gln | M | 78/4 | Spas, atx, dem | Ø | Ø | Ø | 0 | 4 | 3.5 | 2 | ✓ | ✓ | Ø | Ø | norm | NA | T2 | norm |
| 61. | p.Arg486Gln | M | 35/7 | Atx | Ø | Ø | Ø | 0 | 3 | NA | 1 | Ø | Ø | Ø | Ø | norm | Ø | mnl | NA |
| 63. | p.Arg486Gln | F | 67/2 | Spas, atx, dem | Ø | Ø | Ø | 2 | 5 | 2 | 1 | Ø | Ø | ✓ | ✓ | norm | Ø | NA | Ba |
| 64. | p.Arg486Gln | F | 36/9 | Atx | Ø | Ø | Ø | 0 | 5 | 17 | 1 | Ø | Ø | Ø | ✓ | norm | Ø | norm | NA |
